# Multi-Omics Analysis of Hepatopancreatic Responses to Ammonia Nitrogen Stress in Diploid Pacific Oysters (*Magallana gigas*)

**DOI:** 10.3390/ani16172795

**Published:** 2026-09-05

**Authors:** Daowen Qiu, Yuwei Zhang, Lirong Chang, Yanchun Wang, Zan Li, Xiaokai Bao, Xuebo Cui, Cuiju Cui, Guohua Sun, Yanwei Feng, Qiang Wang, Xiaohui Xu, Jianmin Yang, Weijun Wang

**Affiliations:** 1 School of Fisheries, Ludong University, Yantai 264025, China; qdw243955@163.com (D.Q.); 13953696410@163.com (Y.Z.); lizanlxm@163.com (Z.L.); c646175773@163.com (X.C.); cuicuiju@163.com (C.C.); sgh_smile@163.com (G.S.); fywzxm1228@163.com (Y.F.); ldu_fish@163.com (Q.W.); xxh83121@163.com (X.X.); ladderup@126.com (J.Y.); 2 Weihai Changqing Ocean Science & Technology Co., Ltd., Rongcheng 264300, China; changlr@163.com; 3 National Demonstration Center for Experimental Fisheries Science Education, Shanghai Ocean University, Shanghai 201306, China; wycyc0210@163.com; 4 Yantai Haiyu Marine Technology Co., Ltd., Yantai 264000, China; 5 Ministry of Education Key Laboratory of Marine Genetics and Breeding, College of Marine Life Science, Ocean University of China, Qingdao 266003, China; jiujiu564352654@163.com

**Keywords:** *Magallana gigas*, ammonia nitrogen stress, hepatopancreas, transcriptomics, metabolomics

## Abstract

Ammonia nitrogen represents a critical environmental stressor in aquaculture and exerts adverse impacts on the growth, reproduction and survival of shellfish. In the present study, diploid Pacific oysters (*Magallana gigas*) were exposed to ammonia nitrogen stress for 0, 6, and 48 h. We combined histopathological examination, antioxidant enzyme measurements, transcriptome profiling, and metabolome profiling to characterize time-dependent hepatopancreatic responses. Tissue injury became progressively more severe with prolonged exposure. Superoxide dismutase activity increased throughout the experiment, whereas catalase and glutathione peroxidase activities and malondialdehyde content showed an increase followed by a decrease, with no statistically significant differences in malondialdehyde content among the three time points. Transcriptome data revealed alterations in genes associated with ATP-binding cassette transporters, lysosomal function, endocytosis, and autophagy. Metabolic alterations were mainly associated with nucleotide, purine, pyrimidine, and sphingolipid metabolism. Cross-omics integration linked these molecular shifts to transmembrane transport, glutathione metabolism, sulfur-containing amino acid metabolism, and amino acid biosynthesis. The consistency between transcriptomic and metabolomic signals further highlights the central roles of transport processes, antioxidant defense, and metabolic adjustment under ammonia nitrogen exposure. These findings indicate that diploid Pacific oysters respond to ammonia nitrogen stress through coordinated regulation of oxidative stress responses, transmembrane transport, intracellular degradation and clearance, and metabolic reorganization. These findings provide insights into the adaptive mechanisms of bivalves exposed to ammonia nitrogen stress.

## Simple Summary

Ammonia nitrogen accumulation in aquaculture systems can adversely affect the health and survival of cultured shellfish. To investigate the time-dependent response of diploid Pacific oysters to ammonia nitrogen stress, hepatopancreatic samples were collected before exposure (0 h) and after 6 and 48 h of exposure to 10 mg/L ammonia nitrogen. Hepatopancreatic tissue damage became progressively more severe with exposure duration. Antioxidant defenses were rapidly activated at 6 h, whereas prolonged exposure resulted in decreased activities of some antioxidant enzymes despite continued tissue injury. Integrated analyses of gene expression and metabolic profiles indicated coordinated changes in membrane transport, intracellular degradation and recycling, antioxidant defense, and nucleotide, amino acid, and lipid metabolism. These results indicate that diploid Pacific oysters initiate early protective responses to maintain normal cellular function, but these responses may become insufficient under prolonged ammonia nitrogen stress. The findings improve our understanding of the physiological and molecular mechanisms underlying ammonia toxicity in Pacific oysters and may contribute to improved water-quality management and health maintenance in oyster aquaculture.

## 1. Introduction

Ammonia nitrogen is an important component of the nitrogen cycle in aquatic ecosystems, but its excessive accumulation can impair water quality and threaten aquatic organisms. In aquaculture systems, it is a common nitrogenous pollutant and one of the major environmental stressors [1,2]. In aquatic environments, ammonia nitrogen mainly exists as unionized ammonia (NH_3_) and ionized ammonium (NH_4_^+^), and the equilibrium between these two forms is regulated by environmental factors such as temperature, pH, and salinity [3]. Among them, NH_3_ is regarded as the major contributor to ammonia nitrogen toxicity because of its high lipid solubility and ability to freely diffuse across biological membranes [1,4]. With rapid industrial and agricultural development, the discharge of large amounts of nitrogen-containing wastewater has aggravated ammonia nitrogen pollution in aquatic environments [5]. This problem is particularly pronounced in high-density aquaculture systems, where ammonia nitrogen readily accumulates as a result of feed residues, decomposition of excreta, and mineralization of organic matter [6].

In mariculture systems, ammonia nitrogen accumulation not only deteriorates water quality but also directly threatens the physiological functions and survival of cultured animals. Long-term exposure to high concentrations of ammonia nitrogen has been shown to reduce feeding, inhibit growth, and even cause mortality in aquatic animals [7,8,9]. Such exposure can induce multi-level damage by disrupting gill tissue structure, interfering with osmoregulation, and inhibiting key enzyme activities [1,2,10,11]. In addition, ammonia nitrogen stress can trigger oxidative stress and promote the accumulation of reactive oxygen species (ROS), leading to lipid peroxidation and cellular damage [12]. At the immune level, ammonia nitrogen stress may weaken host immune defense and increase the susceptibility of aquatic animals to pathogen infection [1]. In recent years, increasing attention has been paid to the toxic mechanisms of ammonia nitrogen in aquatic animals. In fish, ammonia nitrogen has been shown to impair nervous system function, resulting in abnormal behavior and even mortality [1]. In crustaceans, it can affect molting and energy metabolism [13], whereas in bivalves, it mainly disrupts metabolic homeostasis and immune function by impairing the gills and digestive gland [14]. For example, ammonia nitrogen exposure has been reported to significantly alter the activities of antioxidant enzymes, such as SOD and CAT, induce apoptosis, and activate pathways related to energy metabolism and oxidative stress in oysters [15].

The Pacific oyster (*Magallana gigas*) is one of the most important mariculture bivalves worldwide. Owing to its rapid growth, strong environmental adaptability, and high aquaculture production, it is widely cultured along the northern coastal regions of China and has substantial economic value [16]. As a typical filter-feeding bivalve, Pacific oysters are highly sensitive to environmental changes and respond strongly to aquatic pollutants such as ammonia nitrogen, making them commonly used indicator species for marine environmental monitoring [17,18].

Previous studies have shown that ammonia nitrogen stress can induce oxidative stress, alter immune function, and disrupt energy metabolism in oysters [15,19]. However, the dynamic transcriptomic and metabolomic responses of the hepatopancreas in diploid Pacific oysters under different durations of ammonia nitrogen stress remain poorly understood. As an important organ involved in digestion, metabolism, and detoxification in bivalves, the hepatopancreas is highly responsive to environmental stress. In this study, the hepatopancreas of diploid Pacific oysters was selected as the target tissue, and samples were collected at 0, 6, and 48 h under ammonia nitrogen stress. By integrating histological observation, antioxidant assays, and transcriptomic-metabolomic analyses, this study systematically elucidated the dynamic changes in key genes, differential metabolites, and associated pathways, providing a theoretical basis for understanding the molecular regulatory mechanisms underlying bivalve responses to ammonia nitrogen stress.

## 2. Materials and Methods

### 2.1. Experimental Animals

Two-year-old diploid Pacific oysters were obtained from the Kongtong Island Breeding Base, Yantai, Shandong Province, China. The 120 individuals used for acclimation had a mean wet mass of 184.02 ± 13.11 g and a mean shell length of 66.79 ± 7.91 mm. They were maintained for 7 d before exposure at 25 ± 0.5 °C, pH 8.3 ± 0.2, salinity 32 ± 0.5, and dissolved oxygen above 5 mg/L. Water was renewed and the oysters were fed each day during acclimation. Feed was withheld for 48 h immediately before the ammonia-nitrogen trial.

### 2.2. Experimental Design and Sample Collection

Feed was withheld for 48 h before the ammonia-nitrogen trial, and no feed was provided during the subsequent exposure period to minimize feeding-related fluctuations in water quality and maintain stable exposure conditions. Following acclimation, 60 visibly healthy oysters with intact shells and no apparent signs of disease or injury were randomly selected for the ammonia-exposure experiment. The remaining 60 oysters were not used in any subsequent experiments and were disposed of in accordance with institutional biosafety and environmental management procedures. The selected oysters were randomly allocated to three 160 L tanks, with 20 individuals per tank. A nominal total ammonia nitrogen concentration of 10 mg/L was selected for the exposure experiment based on a preliminary 96 h range-finding trial, in which no mortality was observed at 10 mg/L, whereas mortality occurred at concentrations of 40 mg/L and above. Ammonium chloride (Sinopharm Chemical Reagent Co., Ltd., Shanghai, China) was added to establish an ammonia-nitrogen concentration of 10 mg/L; temperature, pH, salinity, and dissolved oxygen were otherwise maintained at the acclimation values. Sampling was conducted before exposure (C_0h) and after 6 h (N_6h) and 48 h (N_48h). At every time point, three oysters were taken randomly from each tank, giving nine individuals per sampling interval. Samples collected before exposure served as the control group (C_0h), whereas samples collected after 6 and 48 h of exposure constituted the N_6h and N_48h groups, respectively. The three oysters collected from each tank were pooled to constitute one biological replicate, resulting in three biological replicates per group (*n* = 3). Hepatopancreas tissue was removed with pre-cooled instruments, rinsed in phosphate-buffered saline (PBS), immediately frozen in liquid nitrogen, and stored at −80 °C for biochemical and sequencing analyses. Parallel tissue portions were placed in Bouin’s fixative (Ricca Chemical, Arlington, TX, USA) for histology.

### 2.3. Enzyme Activity Assays and Histological Observation

For antioxidant enzyme and MDA assays, hepatopancreatic tissue was homogenized in ice-cold 0.9% physiological saline at a tissue mass-to-solution volume ratio of 1:9 (g/mL). After clean magnetic beads were added, each sample was homogenized at low temperature in an ice-water bath. Homogenates were centrifuged at 3000 rpm and 4 °C for 10 min, and the recovered supernatants were diluted as required for the assays. SOD, CAT, and GSH-Px activities and MDA content were determined with commercial kits (Nanjing Jiancheng Bioengineering Institute, Nanjing, China) in accordance with the supplier’s protocols. Antioxidant enzyme activity and MDA data were obtained from three biological replicates per group (n = 3). Pairwise comparisons among the C_0h, N_6h, and N_48h groups were performed using unpaired *t*-tests implemented in the OmicShare Tools online platform (https://www.omicshare.com/tools accessed on 7 May 2026) during boxplot generation.

For histological processing, hepatopancreas samples were kept in Bouin’s fixative for 24 h and then rinsed with purified water. Following dehydration through increasing ethanol concentrations, the tissues were paraffin embedded, sectioned at 5 μm, and stained with hematoxylin and eosin (H&E). Neutral resin was used to mount the stained sections.

### 2.4. RNA Extraction, Library Construction, and Sequencing

Three independent RNA-seq libraries were prepared for each time point. Each biological replicate represented an equal-mass pool of hepatopancreas tissue from three oysters collected from the same tank. RNA isolation, library preparation, and sequencing were completed by Novogene Co., Ltd. (Beijing, China). Total RNA was isolated with TRIzol reagent (Invitrogen, Waltham, MA, USA) following the manufacturer’s procedure and divided into aliquots for library construction and subsequent qRT-PCR validation. Integrity and quality were examined with an Agilent 2100 Bioanalyzer (Agilent Technologies, Santa Clara, CA, USA). Libraries were generated using the NEBNext^®^ Ultra™ RNA Library Prep Kit for Illumina (New England Biolabs, Ipswich, MA, USA) and sequenced on theIllumina NovaSeq platform (Illumina, Inc., San Diego, CA, USA) [20,21,22].

### 2.5. RNA-Seq Data Processing and Enrichment Analysis

Sequence reads containing adapters, more than 10% undetermined bases, or low-quality base calls were discarded. The retained clean reads were mapped to the Pacific oyster reference genome with HISAT2 (version 2.2.1), after which gene abundance was quantified. DESeq2 (version 1.38.3) was used for pairwise differential expression analysis, with significance defined as |log_2_ fold change| ≥ 1 and *p* ≤ 0.05. Differentially expressed genes (DEGs) were assigned Gene Ontology (GO) annotations and evaluated within the Biological Process, Cellular Component, and Molecular Function domains. Kyoto Encyclopedia of Genes and Genomes (KEGG) enrichment was subsequently used to identify pathways represented among the DEGs.

### 2.6. qRT-PCR Analysis

Ten representative DEGs were examined by quantitative real-time PCR (qRT-PCR) as an independent check of the RNA-seq expression patterns. Primer Premier 5.0 was used to design gene-specific primer pairs (Table 1). EF-1α served as the reference gene because its expression has been shown to be stable [15,19], and relative transcript abundance was calculated with the 2^−ΔΔCt^ method.

### 2.7. Metabolomic Analysis

#### 2.7.1. Metabolite Extraction

Frozen hepatopancreas was pulverized under liquid nitrogen. A 100 mg portion of powder was mixed with 500 μL of 80% methanol-water and vortexed, held on ice for 5 min, and centrifuged at 15,000× *g* for 20 min at 4 °C. The supernatant was diluted with LC-MS-grade water until methanol comprised 53% of the solution. After a second centrifugation under the same speed, temperature, and duration, the clarified supernatant was retained for LC-MS. A pooled quality-control (QC) sample, prepared by combining equal volumes from every experimental extract, was injected throughout the analytical sequence to evaluate instrument stability and data quality.

#### 2.7.2. Data Preprocessing and Differential Metabolite Screening

Compound Discoverer 3.3 was used to process the raw LC-MS files. Candidate features were detected from retention time and mass-to-charge information, and their peak areas were corrected against the first QC injection. Feature extraction used a 5 ppm mass tolerance and a 30% signal-intensity tolerance. Adduct information guided ion integration and quantification, whereas molecular-ion and fragment spectra were used to infer molecular formulas. Putative metabolite identities were assigned by comparison with HMDB, KEGG, and LIPID Maps. Signals also present in a 53% aqueous methanol blank were removed as background. Peak areas were then converted to relative abundance values using the following normalization:

Normalized abundance = sample peak area/(sum of sample peak areas/sum of QC1 peak areas)

Features with a coefficient of variation above 30% across QC samples were excluded before the final identification and relative-quantification tables were generated. Downstream processing was performed in Linux with R (version 3.4.3) and Python (version 2.7.6). The metaX workflow was used for data transformation, multivariate analysis, and calculation of variable importance in projection (VIP). Group-wise differences in individual metabolites were tested with Student’s *t*-test. Metabolites satisfying both VIP > 1 and *p* < 0.05 were classified as differentially abundant.

### 2.8. Integrated Transcriptomic and Metabolomic Analysis

DEGs and DEMs identified in the three pairwise comparisons were independently mapped to the KEGG database. Pathways containing both DEGs and DEMs were considered shared pathways between the transcriptomic and metabolomic datasets. The corresponding genes and metabolites within these shared pathways were subsequently extracted, and their gene–pathway–metabolite associations were visualized using a Sankey diagram. The integrated analysis was performed at the pathway level to identify biological processes showing coordinated transcriptional and metabolic responses during ammonia nitrogen exposure.

## 3. Results

### 3.1. Histological Observation

**Figure 1:**
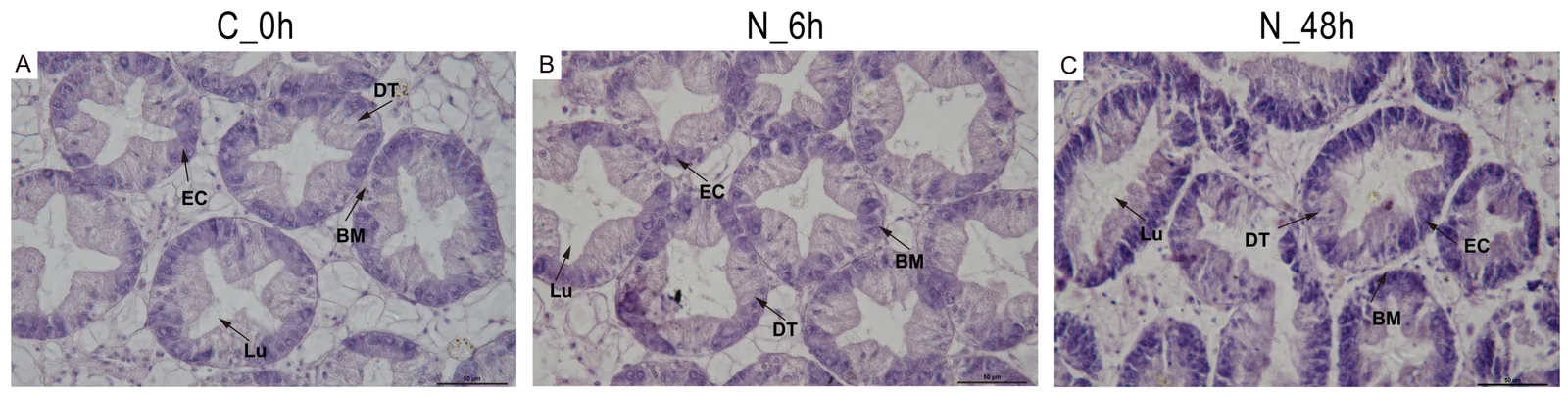
Effects of ammonia nitrogen exposure on hepatopancreatic tissue structure. (**A**) C_0h; (**B**) N_6h; and (**C**) N_48h. DT: digestive tubule; Lu: lumen; EC: epithelial cell; BM: basement membrane. Scale bars = 50 μm.

Histological sections showed that the hepatopancreas of oysters in the C_0h group maintained a relatively intact structure. The digestive tubules were regularly shaped, with clear lumens, compact epithelial cell arrangement, and distinct outer boundaries (Figure 1A). After 6 h of ammonia nitrogen stress, structural abnormalities appeared in the digestive tubules of the N_6h group, including partial lumen dilation, irregular tubule morphology, loosened epithelial cell arrangement, and localized cell swelling or disorganization (Figure 1B). After 48 h of stress, tissue damage was further aggravated in the N_48h group, as indicated by marked disorganization of digestive tubules, more pronounced lumen dilation, irregular epithelial cell arrangement, and local tissue fragmentation or epithelial cell shedding. The outer boundaries of the digestive tubules also became blurred (Figure 1C). Overall, qualitative histological observations showed more pronounced hepatopancreatic alterations at 48 h than at 0 and 6 h.

### 3.2. Changes in Hepatopancreatic Antioxidant Parameters

**Figure 2:**
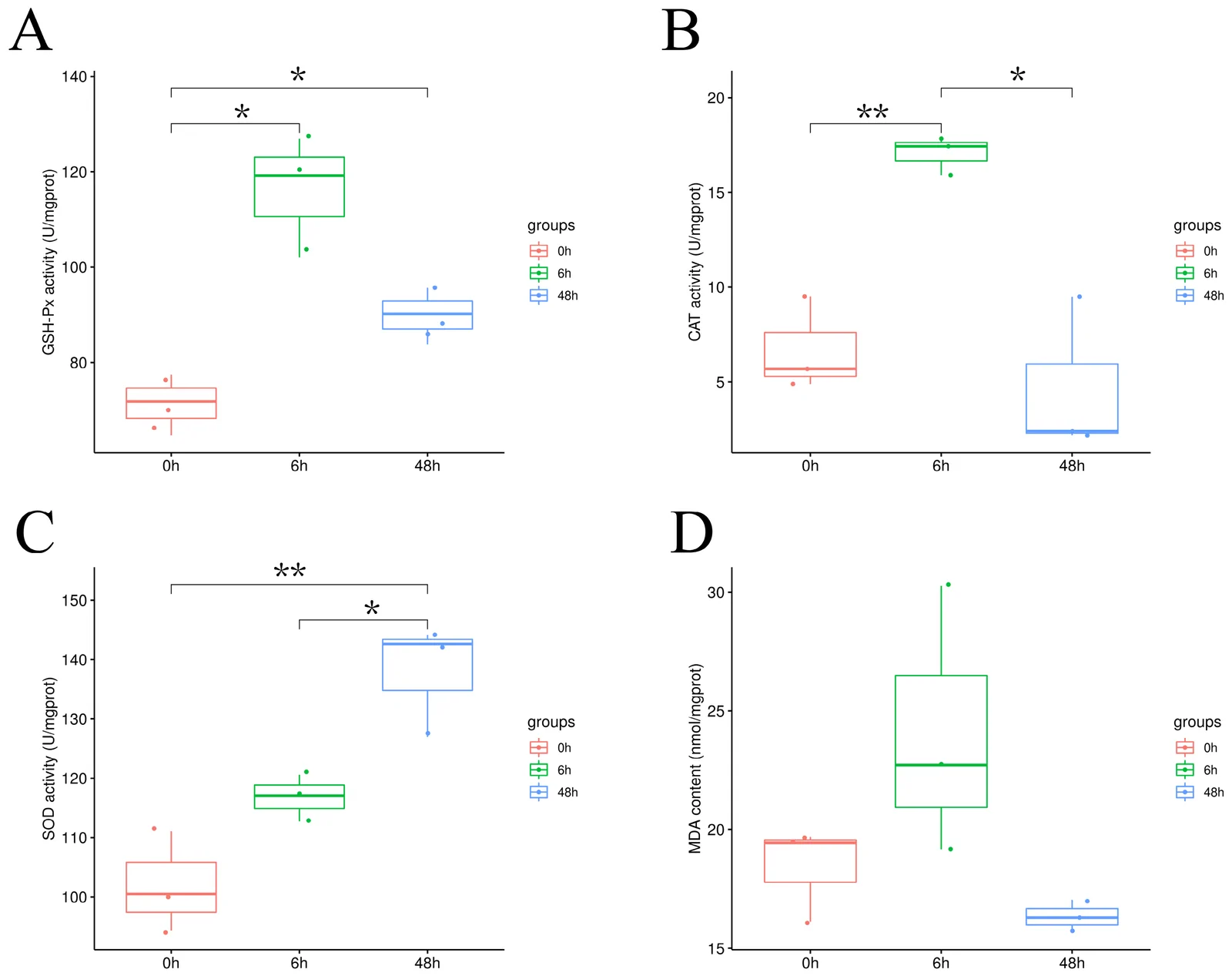
Effects of ammonia nitrogen exposure on antioxidant parameters in the hepatopancreas of diploid Pacific oysters. (**A**) GSH-Px activity; (**B**) CAT activity; (**C**) SOD activity; and (**D**) MDA content. Data are presented as box plots with individual values overlaid. Each point represents one biological replicate, with three biological replicates per group (n = 3). Asterisks indicate significant differences between groups: * *p* < 0.05, ** *p* < 0.01.

Ammonia exposure produced distinct temporal patterns in hepatopancreatic antioxidant indices (Figure 2). GSH-Px and CAT activities were significantly elevated at 6 h relative to 0 h, while SOD activity increased over the exposure period. MDA content showed a numerical increase at 6 h followed by a decrease at 48 h; however, the differences in MDA content among the three time points were not statistically significant.

### 3.3. Transcriptome Sequencing Results

RNA-Seq was performed on diploid Pacific oyster samples, and the sequencing statistics are presented in Table 2. Based on the mapping results, functional annotation was further performed against the NR, NT, Swiss-Prot, GO, and KO databases.

### 3.4. Analysis of DEGs

**Figure 3:**
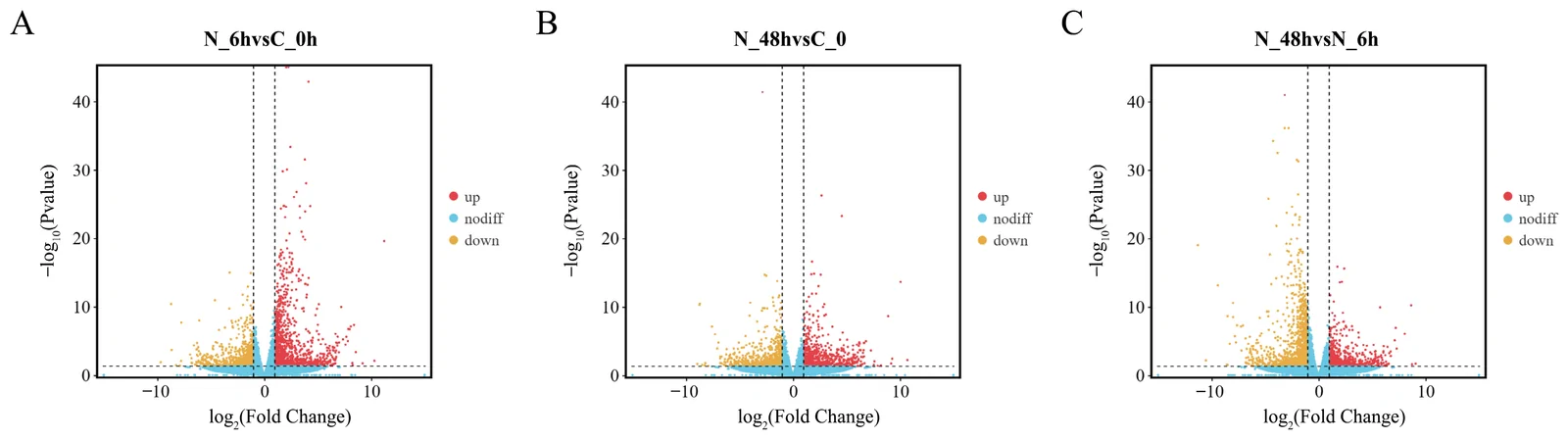
Volcano plots of DEGs in different comparison groups. (**A**) N_6h vs. C_0h; (**B**) N_48h vs. C_0h; and (**C**) N_48h vs. N_6h. Red, orange, and blue dots represent upregulated, downregulated, and non-differentially expressed genes, respectively. The vertical dashed lines indicate log_2_(fold change) thresholds of −1 and 1, and the horizontal dashed line indicates the significance threshold of *p* = 0.05.

**Figure 4:**
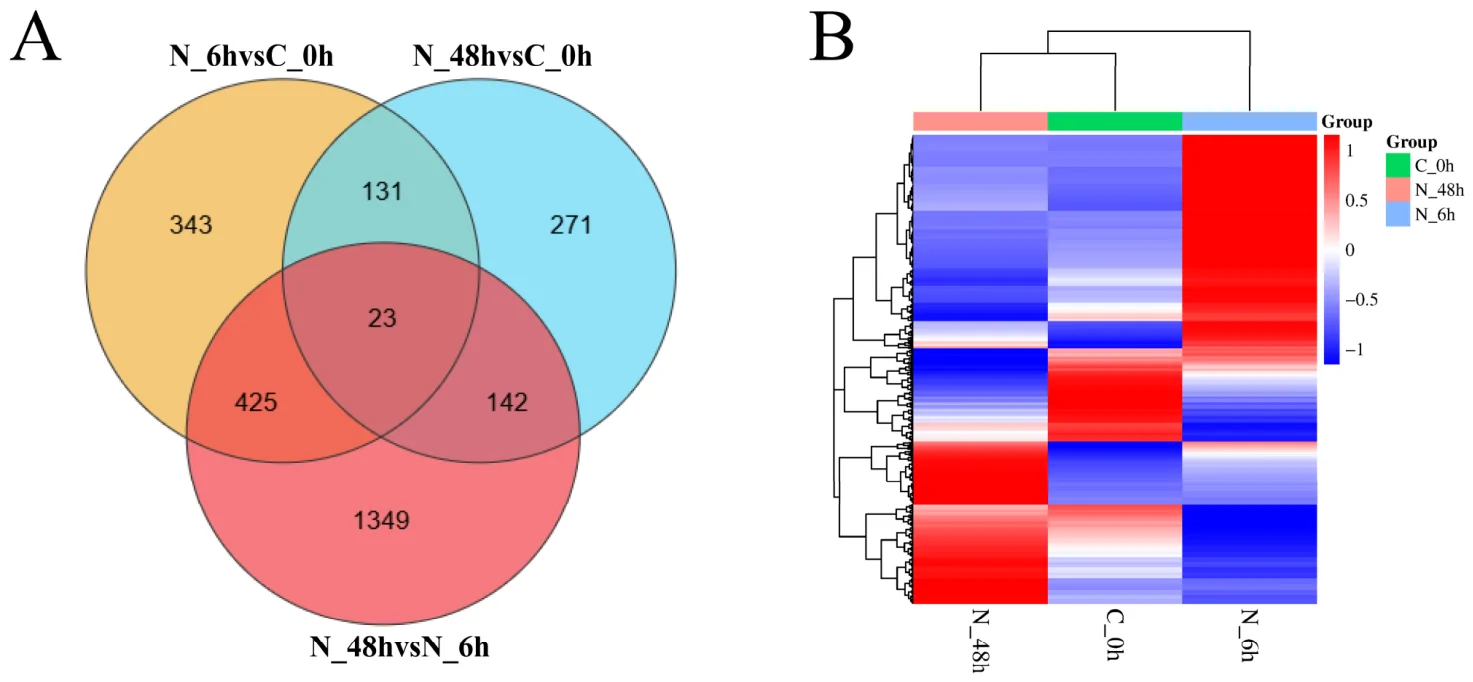
Venn diagram and hierarchical clustering heatmap of DEGs in different comparison groups. (**A**) Venn diagram showing the shared and unique DEGs among the N_6h vs. C_0h, N_48h vs. C_0h, and N_48h vs. N_6h comparisons. (**B**) Hierarchical clustering heatmap showing the expression patterns of DEGs in the C_0h, N_6h, and N_48h groups.

The N_6h vs. C_0h comparison yielded 922 differentially expressed genes (DEGs), of which 316 were upregulated and 606 were downregulated (Figure 3A). At 48 h, 567 DEGs were detected relative to C_0h, including 335 upregulated and 232 downregulated genes (Figure 3B). The N_48h vs. N_6h comparison contained 1939 DEGs, with 770 upregulated and 1169 downregulated genes (Figure 3C). Twenty-three DEGs were shared by all three comparisons (Figure 4A), whereas hierarchical clustering separated the groups on the basis of their expression profiles (Figure 4B).

### 3.5. GO and KEGG Functional Enrichment Analysis

**Figure 5:**
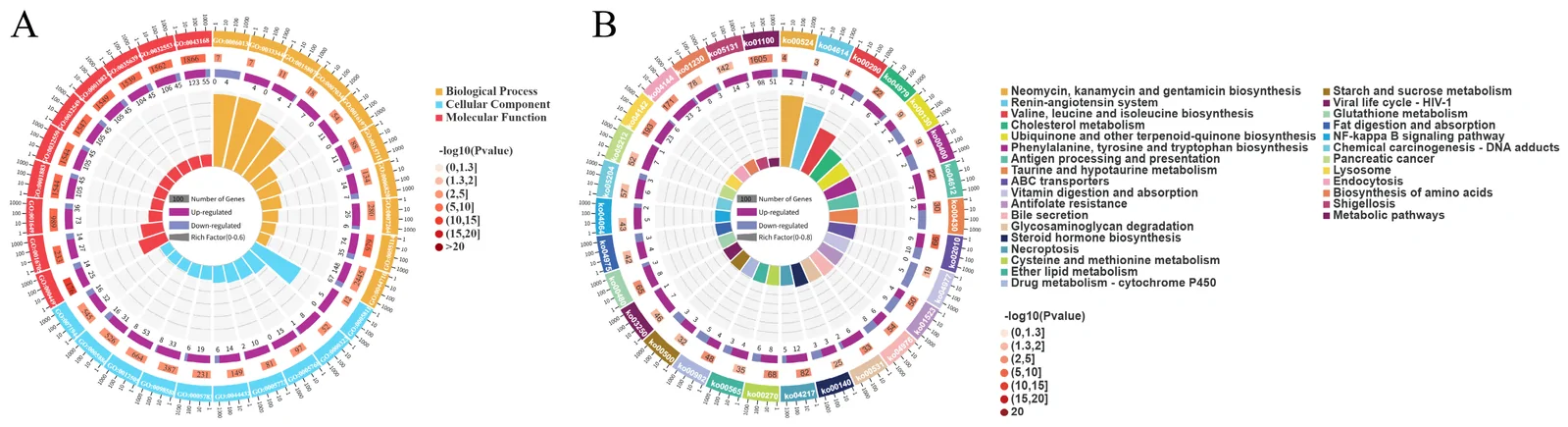
GO and KEGG enrichment analyses of DEGs. (**A**) GO enrichment circle plot. (**B**) KEGG enrichment circle plot.

GO analysis assigned the DEGs to Biological Process (BP), Cellular Component (CC), and Molecular Function (MF), and the 10 most significantly enriched terms in each category were visualized (Figure 5A). Prominent BP terms included oxidation-reduction, vacuolar and endosomal transport, anion transport, and L-amino acid transport. The endomembrane system, organelle-bounding membranes, endosomes, and vacuoles were prominent CC terms, while oxidoreductase activity, monooxygenase activity, and nucleotide binding were represented in MF. KEGG analysis highlighted ABC transporters, lysosome, endocytosis, cysteine and methionine metabolism, and taurine and hypotaurine metabolism (Figure 5B), linking the transcriptional response to membrane transport, intracellular degradation, and redox-related metabolism.

**Figure 6:**
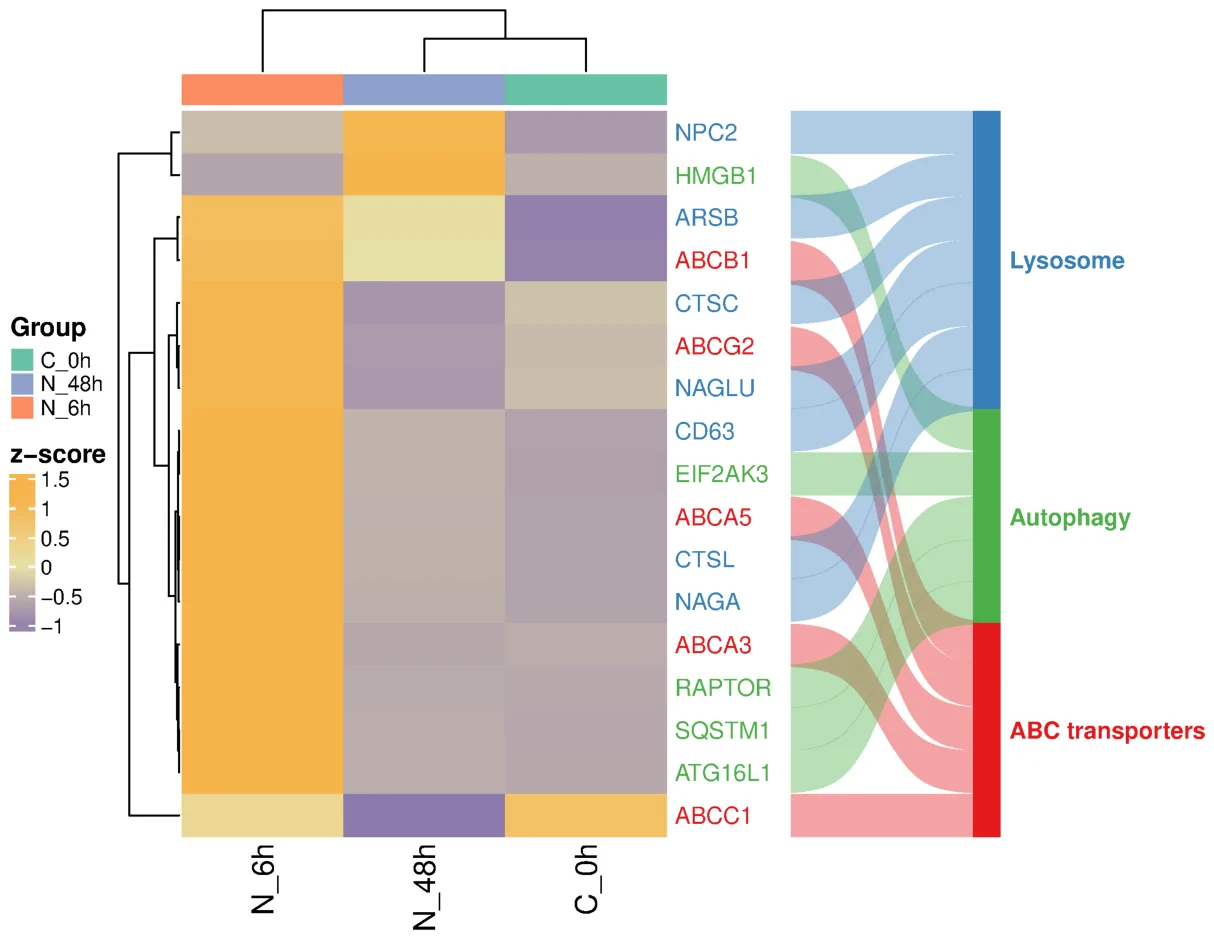
Expression patterns and pathway distribution of DEGs related to ABC transporters, Lysosome, and Autophagy pathways.

Based on the enrichment analysis results, representative DEGs involved in the ABC transporters, Lysosome, and Autophagy pathways were selected for expression pattern analysis (Figure 6). Overall, these genes showed distinct expression patterns across different exposure times, with most genes exhibiting relatively higher expression levels in the N_6h group and altered expression levels in the N_48h group.

### 3.6. qRT-PCR Validation of Representative DEGs

**Figure 7:**
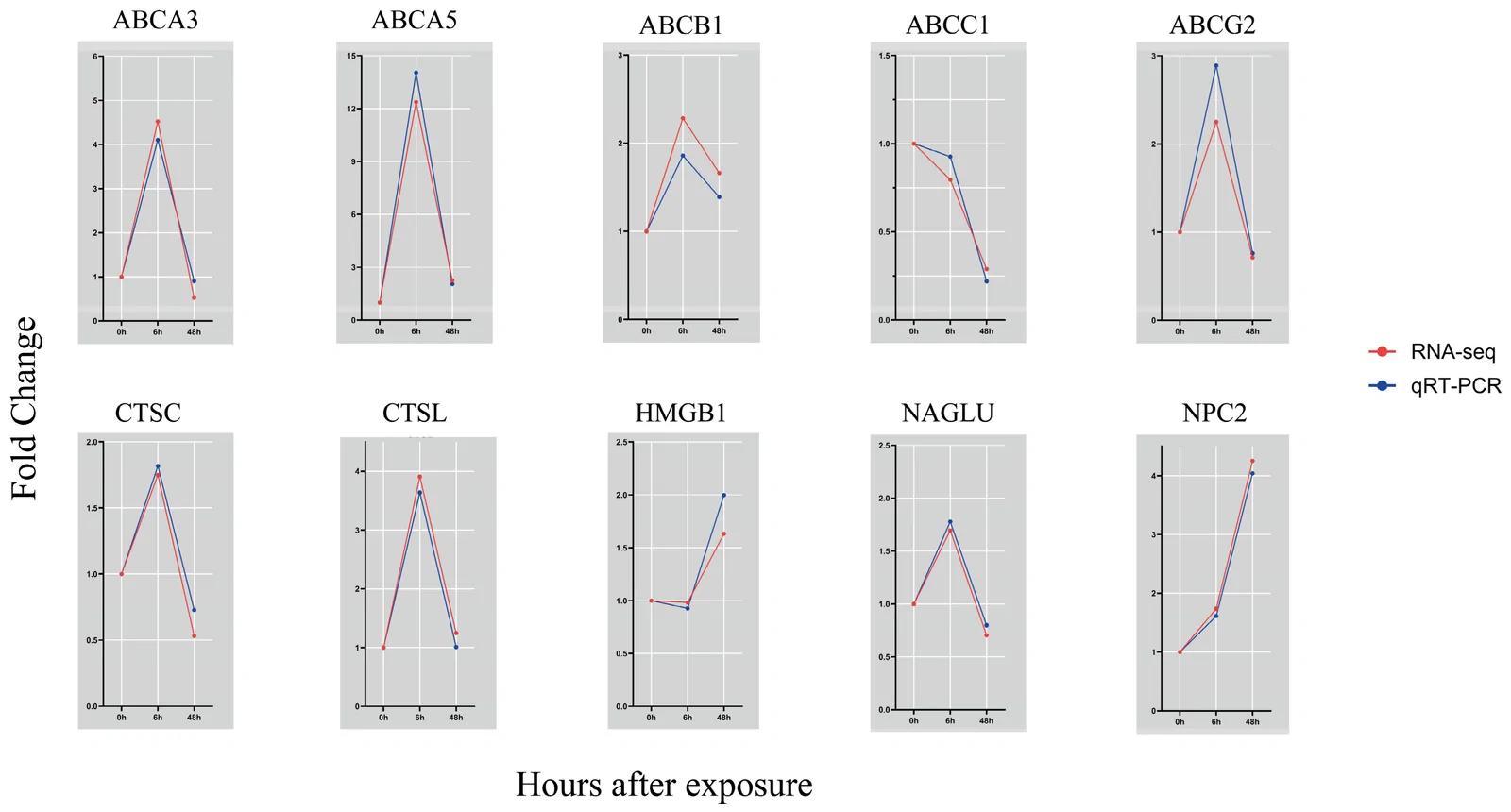
qRT-PCR validation results of representative DEGs. EF-1α was used as the reference gene to normalize gene expression levels. The *x*-axis represents exposure time, and the *y*-axis represents the fold change in relative gene expression.

Expression of 10 representative DEGs was measured by qRT-PCR. For each gene, the direction of change was generally concordant with the corresponding RNA-seq profile (Figure 7), supporting the reliability of the transcriptomic data.

### 3.7. Metabolomic Responses to Ammonia Nitrogen Stress

**Figure 8:**
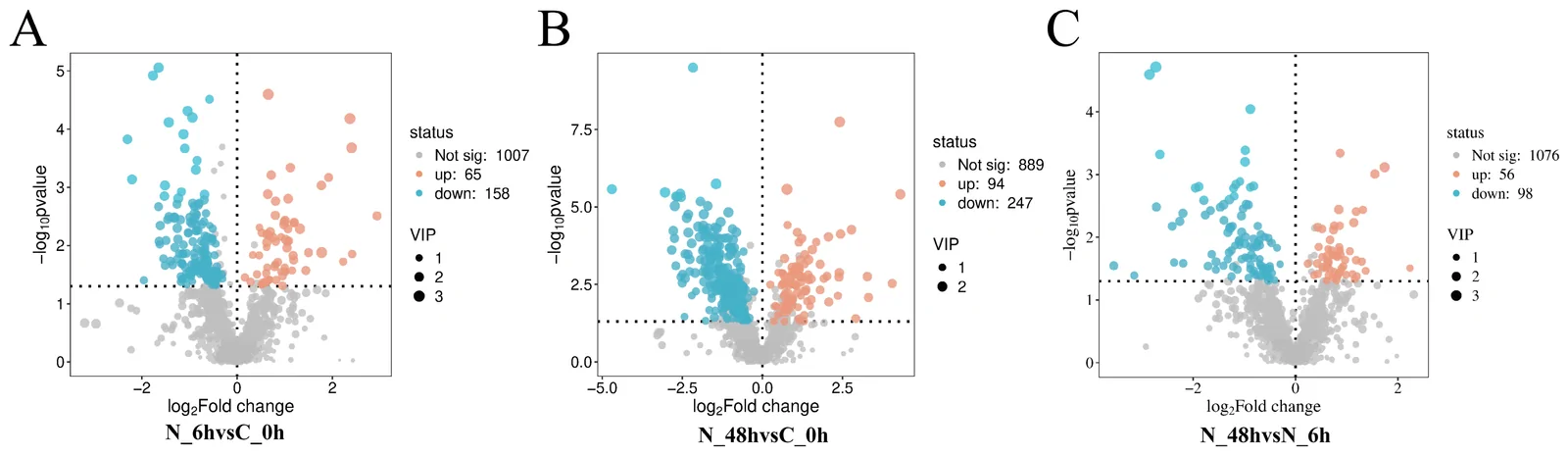
Volcano plot analysis of differential metabolites. (**A**) N_6h vs. C_0h; (**B**) N_48h vs. C_0h; (**C**) N_48h vs. N_6h. Orange dots represent upregulated metabolites (UP), blue dots represent downregulated metabolites (DOWN), and gray dots represent non-significant metabolites (Not sig). The dot size represents the VIP value, with larger VIP values indicating greater contributions to group separation. The vertical dashed line indicates log_2_(fold change) = 0, and the horizontal dashed line indicates the significance threshold of *p* = 0.05.

Metabolite profiles also changed with exposure time. Relative to C_0h, 223 differential metabolites (DEMs) were detected at 6 h, including 65 increases and 158 decreases (Figure 8A). At 48 h, the comparison with C_0h contained 341 DEMs, of which 94 increased and 247 decreased (Figure 8B). Direct comparison of N_48h with N_6h identified 154 DEMs, comprising 56 increases and 98 decreases (Figure 8C). The predominance of decreased features in both comparisons with C_0h and the larger number of DEMs at 48 h indicated progressive remodeling of the hepatopancreatic metabolome.

**Figure 9:**
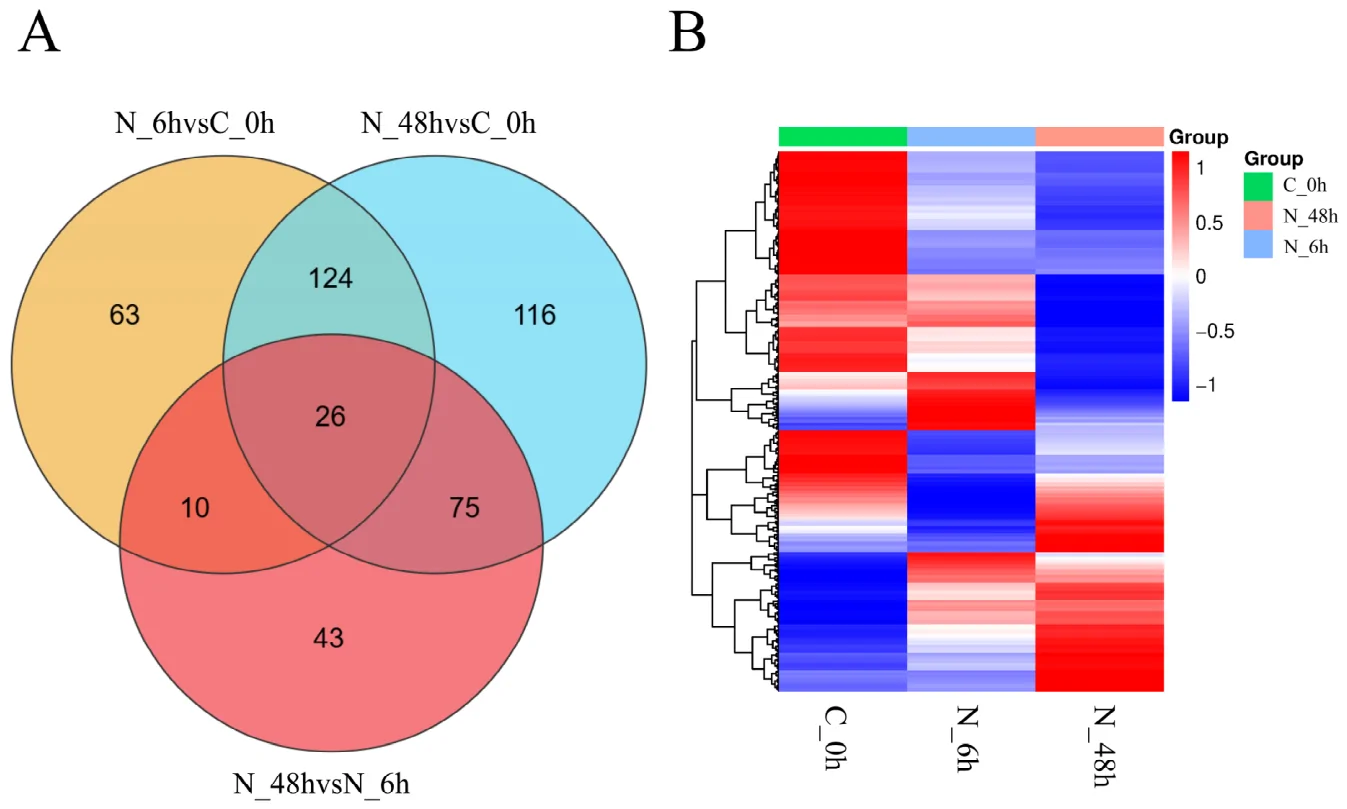
Venn diagram and clustering heatmap of DEMs. (**A**) Venn diagram. (**B**) Clustering heatmap.

The Venn diagram showed the distribution of shared and unique DEMs among the different comparison groups. A total of 26 DEMs were common to all three comparisons (Figure 9A). The clustering heatmap showed a certain degree of separation among samples from different time points based on the abundance patterns of DEMs (Figure 9B).

### 3.8. KEGG Enrichment Analysis of Differential Metabolites

**Figure 10:**
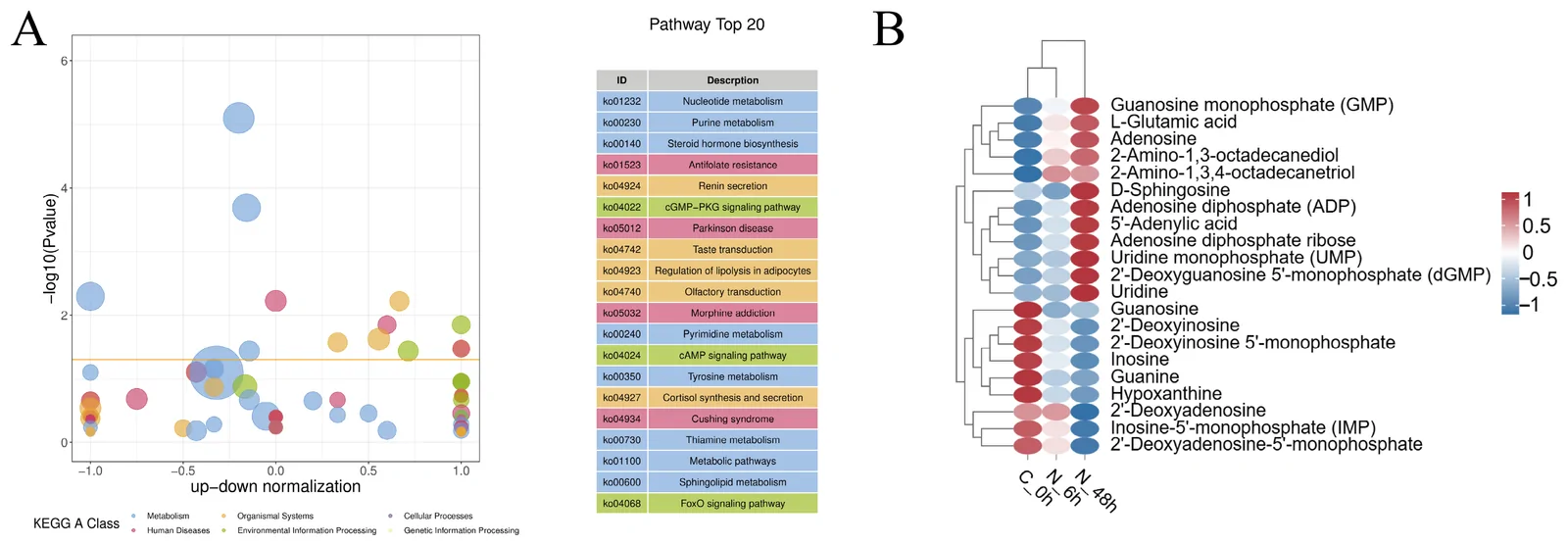
KEGG enrichment analysis of differential metabolites and heatmap of representative metabolites. (**A**) Bubble plot of KEGG pathway enrichment for differential metabolites, showing the top 20 enriched pathways without species-specific pathway filtering. (**B**) Heatmap of the relative abundance of representative differential metabolites. The orange horizontal line in panel A indicates the significance threshold of *p* = 0.05.

KEGG enrichment analysis showed that the differential metabolites were mainly enriched in pathways including nucleotide metabolism, purine metabolism, pyrimidine metabolism, thiamine metabolism, tyrosine metabolism, and sphingolipid metabolism (Figure 10A). Vertebrate-specific terms in the unfiltered enrichment results likely arose from the cross-annotation of shared metabolites to multiple KEGG pathways and were therefore excluded from the biological interpretation. Among these pathways, nucleotide metabolism and purine metabolism showed relatively high enrichment levels, suggesting that ammonia nitrogen stress may markedly affect nucleotide-related metabolism in the hepatopancreas of Pacific oysters. Further heatmap analysis of representative differential metabolites in related pathways revealed clear differences in metabolite abundance among different time points. Metabolites such as GMP, adenosine, ADP, 5′-adenylic acid, UMP, uridine, and dGMP showed higher abundance at 48 h, whereas guanosine, inosine, guanine, hypoxanthine, 2′-deoxyadenosine, IMP, and 2′-deoxyadenosine-5′-monophosphate were relatively decreased at 48 h (Figure 10B). These results indicate that ammonia nitrogen stress altered the abundance of purine-, pyrimidine-, and nucleotide-related metabolites in the hepatopancreas of Pacific oysters, suggesting that nucleic acid metabolism and energy metabolism may be affected.

### 3.9. Integrated Transcriptomic and Metabolomic Pathway Analysis

**Figure 11:**
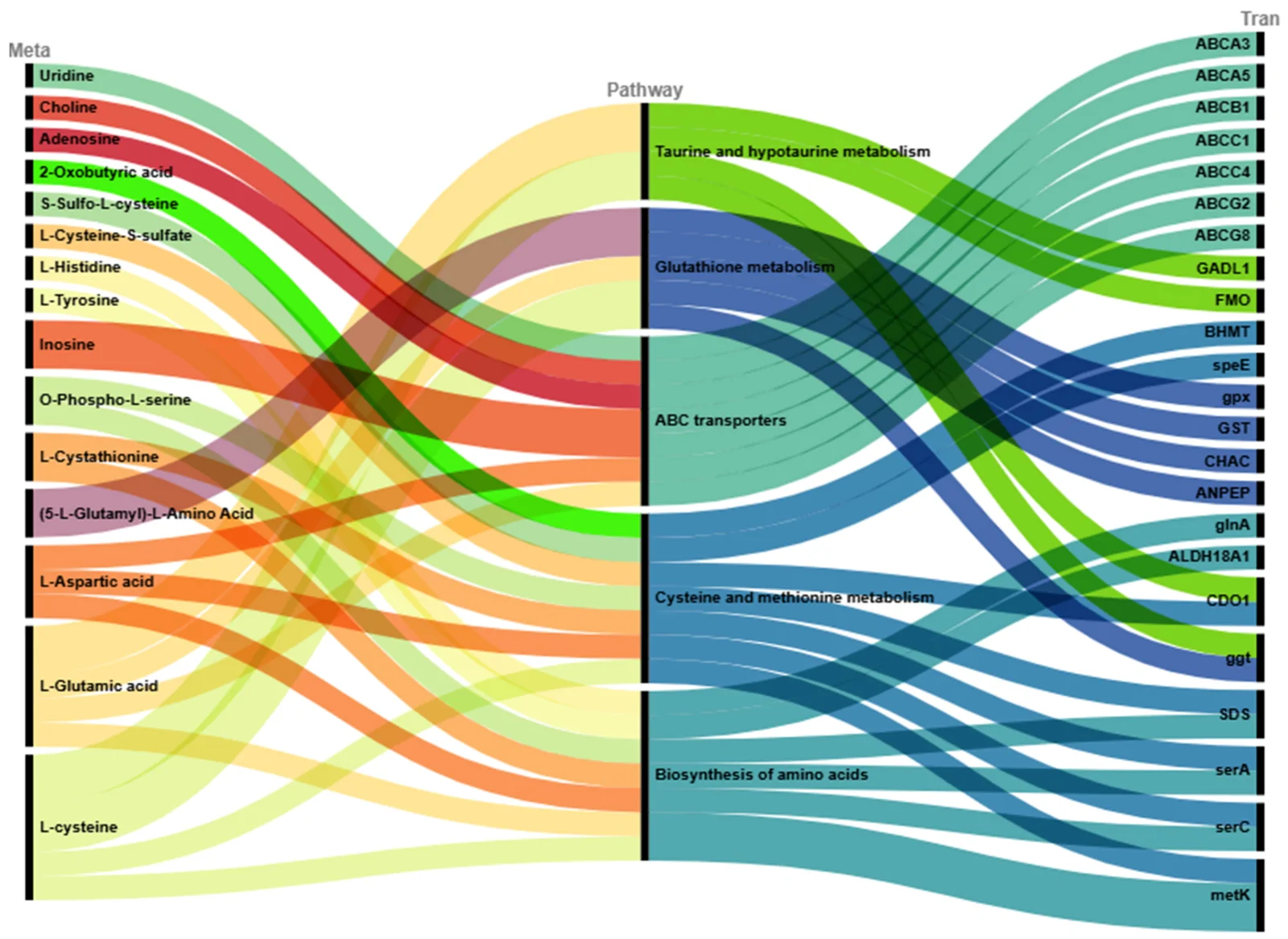
Sankey diagram of integrated KEGG pathway analysis of DEGs and DEMs.

Mapping DEGs and DEMs to KEGG identified common representation in ABC transporters, taurine and hypotaurine metabolism, glutathione metabolism, cysteine and methionine metabolism, and amino acid biosynthesis. The metabolite component included L-glutamic acid, L-aspartic acid, L-cysteine, inosine, and choline, while associated genes included members of the ABC transporter family together with *CDO1*, *GADL1*, *gst*, *gpx*, *ggt*, and *metK* (Figure 11). These shared pathways connect membrane transport with sulfur-containing amino acid metabolism, glutathione-dependent redox processes, and biosynthetic adjustment.

## 4. Discussion

Ammonia nitrogen is a common stressor in high-density aquaculture environments. Previous studies on oysters have shown that acute ammonia nitrogen exposure can induce time- and concentration-dependent changes in hemocyte immune parameters in the Hong Kong oyster (*C. hongkongensis*) and affect metabolic processes related to energy metabolism, osmoregulation, and oxidative stress in gill tissues [23]. In triploid Pacific Oyster, ammonia nitrogen exposure has also been reported to cause hepatopancreatic tissue damage, disrupt antioxidant enzyme activities, and activate lysosome-, phagosome-, and autophagy-related regulatory processes [19]. Therefore, the response of oysters to ammonia nitrogen stress is not limited to antioxidant defense, but involves coordinated regulation at multiple levels, including transmembrane transport, clearance of damaged cellular components, and maintenance of cellular homeostasis.

### 4.1. Oxidative Stress and Tissue Damage Induced by Ammonia Nitrogen Stress

Ammonia nitrogen stress can disrupt the dynamic balance between ROS production and scavenging in aquatic animals, thereby inducing oxidative stress and tissue damage [24,25,26]. SOD serves as the first line of antioxidant defense by catalyzing the dismutation of superoxide anions (O_2_^−^) into H_2_O_2_, while CAT and GSH-Px further remove H_2_O_2_, reducing peroxide-induced damage to cell membranes, proteins, and nucleic acids [25]. MDA, an important end product of lipid peroxidation, is commonly used as an indicator of oxidative damage to cell membranes [27,28]. SOD activity increased at both 6 h and 48 h, indicating that ammonia nitrogen stress continuously activated the primary antioxidant defense in the hepatopancreas of Pacific oysters. This response may enhance the scavenging of superoxide anion radicals through elevated SOD activity [19,25]. The increased CAT and GSH-Px activities at 6 h indicated rapid activation of antioxidant defenses during the early stage of stress, whereas the decline in these activities at 48 h may reflect inhibition or depletion of the antioxidant system under prolonged ammonia nitrogen exposure [15,25]. Specifically, the sustained increase in SOD activity may reflect the continuous production of superoxide anions, whereas the decline in CAT and GSH-Px activities at 48 h could result from enzyme inactivation by excess H_2_O_2_ or feedback inhibition under prolonged ammonia nitrogen exposure. Although MDA content showed a numerical decrease at 48 h compared with 6 h, this difference was not statistically significant. Meanwhile, qualitative histological observations showed more pronounced hepatopancreatic alterations at 48 h. Therefore, the numerical decrease in MDA content should not be interpreted as evidence of alleviated oxidative injury. Similar patterns have also been reported in tetraploid Pacific oysters exposed to ammonia nitrogen [15]. However, the histopathological assessment was qualitative; therefore, these findings should be interpreted as representative morphological observations rather than statistically quantified differences in tissue damage. Within this qualitative framework, the more evident lumen dilation, epithelial disorganization, and tissue fragmentation observed at 48 h were consistent with more pronounced hepatopancreatic injury during longer exposure. Overall, the enzyme activity and histological results suggest that diploid Pacific oysters mainly enhance antioxidant enzyme activities for early stress defense, but with prolonged exposure, the antioxidant system may fail to fully counteract ammonia-induced cellular stress, ultimately leading to aggravated hepatopancreatic damage. Metabolomic results further showed that ammonia nitrogen stress significantly affected nucleotide metabolism, purine metabolism, and pyrimidine metabolism in the hepatopancreas of Pacific oysters. Nucleotides and their derivatives are not only involved in nucleic acid synthesis, but are also closely related to cellular energy transfer and metabolic regulation under stress conditions [29]. In this study, the abundance of multiple nucleotide-related metabolites changed markedly at 48 h, suggesting that prolonged ammonia nitrogen stress indicating remodeling of nucleic acid and energy metabolism in hepatopancreatic cells. These changes were consistent with aggravated tissue damage and altered antioxidant enzyme activities, supporting metabolic disturbance as an important manifestation of cellular damage and stress adaptation under ammonia nitrogen stress.

### 4.2. ABC Transporter-Mediated Transmembrane Transport Response

ABC transporters are important transmembrane efflux systems through which bivalves respond to environmental stress. They transport xenobiotic toxicants, conjugated metabolites, and lipid-related substrates in an ATP-dependent manner, thereby reducing intracellular toxic burden and maintaining cellular homeostasis. Previous studies have shown that ABCB and ABCC transporters are important components of the multixenobiotic resistance (MXR) system in bivalves and can form an “environment–tissue barrier” in environmentally exposed tissues such as gills [30]. In addition, *ABCB1* and ABCC transporters in Thick shell mussel (*Mytilus coruscus*) participate in Benzo(α)pyrene detoxification, while Pacific oysters mainly reduce toxic damage under diarrhetic shellfish poisoning (DSP) toxin exposure through ABC transporters and toxin esterification, suggesting that ABC transporters play a general role in bivalve defense against toxic stress [31,32]. Studies on green mussels (*Perna viridis*) have also shown that DSP toxin exposure can induce stage-specific responses of ABCB and ABCC transporters, indicating that ABC transporters show time-dependent characteristics in bivalve defense against toxic stress [33]. In this study, *ABCA3*, *ABCA5*, *ABCB1*, and *ABCG2* were generally upregulated during the early stage of ammonia nitrogen stress, whereas most of these genes decreased or returned toward lower levels at 48 h; *ABCC1*, however, remained generally low. This pattern indicates that the ABC pathway response showed clear stage-specific characteristics. The early upregulation of *ABCA3* and *ABCA5* may be related to membrane lipid transport, membrane structural remodeling, and maintenance of cellular homeostasis. *TgABCA3* in blood clams (*Tegillarca granosa*) has been reported to participate in active Cd ion transport and detoxification, further supporting the potential role of ABCA3-type transporters in bivalve toxic stress responses [34]. Therefore, the upregulation of multiple ABC members at 6 h indicates that hepatopancreatic cells of diploid Pacific oysters activated transmembrane transport-related defense processes, which may help transport lipid metabolites, conjugated metabolites, or oxidative damage-related products generated under ammonia nitrogen stress, thereby indirectly reducing cellular toxic burden. The decrease in most ABC genes at 48 h, together with decreased CAT and GSH-Px activities and aggravated hepatopancreatic damage, may reflect limitations in energy supply, membrane integrity, or transport system capacity under prolonged stress. As stress duration increased, transmembrane transport alone may have been insufficient to maintain cellular homeostasis, and the autophagy-lysosome system may have been further required for the degradation and clearance of damaged components. In the integrated transcriptomic and metabolomic analysis, ABC transporters were enriched among both DEGs and DEMs, further supporting the involvement of ABC transporters not only participate in harmful substance efflux, but also be involved in metabolite transmembrane transport and cellular homeostasis maintenance under ammonia nitrogen stress. Oxidative damage products, lipid-related metabolites, or conjugated metabolites generated during ammonia nitrogen stress may need to be exported or redistributed through transport systems; therefore, the stage-specific response of ABC transporters may be closely related to metabolite processing and toxic burden reduction [30,32,33].

### 4.3. Intracellular Clearance and Homeostasis Maintenance Involving the Autophagy-Lysosome Pathway

In this study, both autophagy and lysosome pathways responded to ammonia nitrogen stress, supporting the involvement of an intracellular damage-clearance system centered on the autophagy-lysosome pathway. Autophagy is mainly responsible for recognizing, engulfing, and transporting damaged proteins, abnormal organelles, and intracellular waste, whereas lysosomes mediate final degradation and material recycling. Previous studies have identified a complete autophagy pathway in Pacific oysters and confirmed the presence of autophagy in oyster hemocytes, where it may participate in cellular homeostasis maintenance and immune responses [35,36]. Previous studies on aquatic animals have also shown that the autophagy-lysosome system is an important subcellular target of environmental stress and pollutants. Hypoxia/reoxygenation, pollutant exposure, and ammonia nitrogen stress in bivalves can all affect lysosomal stability and autophagy-related responses [37,38,39]. Ammonia nitrogen exposure in Manila clams (*Ruditapes philippinarum*) also found that it reduced lysosomal integrity and caused tissue damage, indicating that the lysosomal system may be one of the important subcellular targets of ammonia nitrogen toxicity in bivalves [38].

From the perspective of the autophagy pathway, *RAPTOR*, *EIF2AK3*, *SQSTM1*, and *ATG16L1* all showed upregulation trends at 6 h, indicating early transcriptional regulation of autophagy-related processes in the oyster hepatopancreas. These changes may be accompanied by alterations in endoplasmic reticulum stress, selective substrate recognition, and autophagosome formation. *RAPTOR* is an important component of mTORC1, and mTOR is a key upstream pathway regulating autophagy. Studies on ammonia nitrogen stress in yellow catfish (*Pelteobagrus fulvidraco*) have shown that the SLC38A9-mTOR axis is involved in ammonia-induced autophagy regulation, and that enhanced autophagy can alleviate ammonia-induced oxidative stress, inflammation, and apoptosis [40,41]. The upregulation of *EIF2AK3/PERK* is consistent with increased endoplasmic reticulum stress and proteostasis pressure during the early stage of acute stress. As a selective autophagy receptor, *SQSTM1/p62* can mediate the delivery of ubiquitinated substrates to autophagosomes. *ATG16L1* is an important factor in autophagosome formation, and *CgATG16L1* in Pacific oysters has been shown to promote autophagosome and autolysosome formation and participate in antibacterial immunity [42]. In addition, studies on LC3-mediated mitophagy in Pacific oysters have shown that, under CCCP or *Vibrio splendidus* stimulation, oyster hemocytes exhibit colocalization of LC3 with lysosomes and mitochondria with lysosomes, indicating that autophagy/mitophagy may participate in damaged organelle clearance and immune defense [43]. Therefore, the upregulation of these autophagy-related genes at 6 h supports the activation of protective responses related to proteostasis regulation, selective substrate recognition, and damaged component clearance in oyster hepatopancreatic cells.

Changes in the lysosome pathway further support the involvement of the autophagy-lysosome system in the response to ammonia nitrogen stress. *ARSB*, *NAGA*, *CTSL*, *CD63*, *NPC2*, *NAGLU*, and *CTSC* all showed upregulation trends at 6 h, supporting the concurrent transcriptional regulation of lysosomal degradation-related processes alongside autophagic substrate recognition and autophagosome formation. Among these genes, *ARSB*, *NAGA*, and *NAGLU* are not directly involved in ammonia nitrogen metabolism, but more likely reflect an increased demand for the degradation and recycling of glycoconjugates and structural components after ammonia-induced oxidative stress, tissue damage, and lysosomal pressure [19,44,45,46]. Similarly, one study on immune stimulation in pearl oysters (*Pinctada fucata martensii*) found that butyrate treatment activated the lysosome pathway and upregulated lysosomal genes related to carbohydrate and lipid metabolism, such as *ARSB* and *NAGA*. Under long-term high-pH stress in Chinese mitten crabs (*Eriocheir sinensis*), the glycosaminoglycan degradation pathway containing NAGLU was also enriched, suggesting that glycoconjugate degradation may be an important process in aquatic animals responding to environmental stress and tissue remodeling [45,46]. In addition, *CTSL* and *CTSC* are lysosomal proteases involved in the degradation of abnormal proteins and phagocytic substrates. *CgCTSL1* in Pacific oysters has been shown to be highly expressed in phagocytic hemocytes and to participate in proteolysis and cellular immunity, while *CTSC* in razor clams (*Sinonovacula constricta*) also responds to bacterial stimulation, suggesting that cathepsin genes are closely associated with immune clearance in bivalves [47,48]. *CD63* is related to late endosome/phagosome maturation and lysosomal membrane trafficking. Previous studies have shown that *CD63H* in Pacific oysters participates in the formation of pathogen-containing phagosomes and hemocyte immune responses [49]. Therefore, the upregulation of *CD63* and other lysosome-related genes at 6 h supports enhanced intracellular material transport and lysosomal degradation processes under acute ammonia nitrogen stimulation to clear damaged components or external stimuli.

Notably, most autophagy- and lysosomal degradation-related genes decreased at 48 h compared with 6 h, whereas *HMGB1* and *NPC2* continued to increase. *HMGB1* is both a damage-associated molecule and a regulator of autophagy. Studies in fish have shown that ROS can promote *HMGB1*-mediated autophagy, and *HMGB1* paralogs are also involved in autophagy regulation in fish [50,51]. *NPC2* is mainly involved in cholesterol transport in late endosomes/lysosomes. In Asiatic hard clam (*Meretrix meretrix*) exposed to harmful algae, lysosome-related genes such as *NPC2* were significantly upregulated and were considered to be associated with metabolic remodeling and responses to external stimuli [52]. Therefore, the sustained increase in *HMGB1* and *NPC2* at 48 h suggests that the overall decline of autophagy- and lysosome-related genes at the later stage does not necessarily indicate complete recovery from stress, but may reflect enhanced damage signaling and persistent pressure on membrane lipid homeostasis.

Overall, the autophagy-lysosome pathway exhibited a clear stage-dependent response under ammonia nitrogen stress. At 6 h, genes related to autophagy regulation, damaged component recognition, phagosome transport, and lysosomal degradation and recycling generally showed upregulation trends, suggesting rapid mobilization of intracellular clearance and degradation processes during the early stage. At 48 h, most autophagy- and lysosomal degradation-related genes decreased compared with 6 h, whereas *HMGB1* and *NPC2* remained elevated, suggesting that the later response may shift from early clearance processes toward persistent damage signaling and increased pressure on membrane lipid homeostasis. This pattern was consistent with the progressive aggravation of hepatopancreatic tissue damage, supporting the coordinated involvement of the autophagy–lysosome system and ABC transporter-related transmembrane transport in the adaptive response of Pacific oysters to ammonia nitrogen stress, playing important roles in damaged component clearance, metabolite processing, and cellular homeostasis maintenance. In terms of temporal coordination, the concurrent upregulation of several ABC transporter-, autophagy-, and lysosome-related genes at 6 h indicated that transmembrane transport and intracellular clearance processes were jointly engaged during the early response. At 48 h, the expression of most of these genes declined, whereas HMGB1 and NPC2 remained elevated, which was consistent with a transition from broad early activation to a more selective and persistent response associated with damage signaling and membrane lipid homeostasis. In addition, integrated analysis showed that DEGs and DEMs were commonly enriched in Glutathione metabolism, Cysteine and methionine metabolism, and Biosynthesis of amino acids. Glutathione metabolism is an important component of cellular antioxidant defense, and cysteine is a key precursor for glutathione synthesis [53,54]. Changes in metabolites such as L-cysteine and L-glutamic acid, together with changes in genes such as *gst*, *gpx*, *ggt*, and *metK*, support the involvement of sulfur-containing amino acid and glutathione metabolism in redox homeostasis during ammonia nitrogen stress in Pacific oysters. These results are consistent with changes in antioxidant enzyme activities and the response of the autophagy-lysosome pathway, indicating that cellular homeostasis maintenance depends not only on the clearance of damaged components but also on remodeling of the antioxidant metabolic network.

Nevertheless, because time-matched unexposed control groups were not included, the potential contributions of prolonged fasting and other time-dependent factors to the observed responses cannot be completely excluded. Future studies should include parallel ammonia-free control groups sampled at corresponding time points to distinguish ammonia-specific effects more clearly.

## 5. Conclusions

Overall, the response of diploid Pacific oysters to ammonia nitrogen exposure was characterized by the coordinated regulation of antioxidant defense, ABC transporter-mediated transmembrane transport, autophagy–lysosome-related intracellular clearance, and metabolic remodeling. These processes were strongly engaged during the early response, whereas longer exposure was accompanied by persistent hepatopancreatic damage and altered cellular homeostasis. However, the present study examined only two post-exposure time points, 6 and 48 h, which limited the temporal resolution of the observed responses. Future studies incorporating additional sampling time points are needed to characterize the dynamic transitions among these response processes more precisely.

## Figures and Tables

**Table 1:** Primers used for quantitative RT-PCR validation.

Gene Name	Forward Primer (5′–3′)	Reverse Primer (5′–3′)
*EF–1α*	AGTCACCAAGGCTGCACAGAAAG	TCCGACGTATTTCTTTGCGATGT
*ABCA3*	CGCTATGTCTTACGGGGGTC	AGCACCGTATCCACCAACAG
*ABCA5*	TGCCCTCACCAACTCCATTC	CGCCATTGTTGAACCGGTTT
*ABCB1*	AAAACGGCGACTGGTTTTCG	TATAGGAACCCCGCCCAGAA
*ABCC1*	GCTGCCATCACTTATGCTGC	CTTGCACCAGCCAGTGTTTC
*ABCG2*	CGTGATACAGGAGCTCGGAC	CAGGACAGGAGGGGAGATGA
*CTSC*	AAGCAGAGTCTGTGGAAGGC	AACTGGTGCAGGTTTGGGAA
*CTSL*	TCTGGAGTCCGCACAGAAAC	GCGACGACTTTCCTCTTCCA
*HMGB1*	ATGGTCTGTGGCAGAGGTTG	GGTTTGGCTTTCTTGGCTGG
*NAGLU*	TCTCTCTGGCCGCACAATTT	TTGCTTCCAGAATCCGCCAT
*NPC2*	GGCACTCTCACAACTGTGGA	AGTGAAGGTTGCCCTCAGTG

**Table 2:** Summary statistics of transcriptome sequencing data.

Samples	Raw Reads	Clean Reads	Q20 (%)	Q30 (%)	GC (%)	Mapping Rate (%)
C_0h_1	43,895,268	42,757,940	99.29	97.79	43.25	61.69
C_0h_2	40,067,446	38,966,892	99.36	97.96	43.01	61.08
C_0h_3	41,251,156	40,063,214	99.29	97.74	43.09	61.69
N_6h_1	46,183,810	45,020,052	99.38	98.03	42.58	62.59
N_6h_2	50,168,410	47,756,162	99.39	98.07	42.86	61.91
N_6h_3	41,252,586	39,560,100	99.27	97.71	43.02	61.36
N_48h_1	44,641,682	43,351,682	99.37	98.01	43.02	62.13
N_48h_2	44,558,520	43,107,334	99.39	98.06	43.18	61.31
N_48h_3	41,950,740	40,587,848	99.38	98.05	43.08	61.3
Average	43,774,402	42,352,358	99.35	97.94	43.01	61.67

## Data Availability

The original contributions of this study are presented in this article. Further inquiries can be directed to the corresponding author.

## Abbreviations

The following abbreviations are used in this manuscript:

SOD	superoxide dismutase
CAT	catalase
GSH-Px	glutathione peroxidase
MDA	malondialdehyde
RNA-seq	RNA sequencing
LC-MS	liquid chromatography–mass spectrometry
LIPID MAPS	Lipid Metabolites and Pathways Strategy
NR	non-redundant protein sequence database
KO	KEGG Orthology
GC	guanine–cytosine content
DEG	differentially expressed gene
DEM	differential metabolite
QC	quality control
VIP	variable importance in projection
GO	Gene Ontology
KEGG	Kyoto Encyclopedia of Genes and Genomes
ROS	reactive oxygen species
qRT-PCR	quantitative real-time polymerase chain reaction
